# MicroRNA-200a confers chemoresistance by antagonizing TP53INP1 and YAP1 in human breast cancer

**DOI:** 10.1186/s12885-017-3930-0

**Published:** 2018-01-12

**Authors:** San-Jian Yu, Liu Yang, Qi Hong, Xia-Ying Kuang, Gen-Hong Di, Zhi-Ming Shao

**Affiliations:** Breast Cancer Institute, Department of Breast Surgery, Cancer Hospital/Cancer Institute, Shanghai Medical College, Institutes of Biomedical Sciences, Fudan University, #270 Dong An Road, Shanghai, 200032 People’s Republic of China

**Keywords:** microRNA, Breast cancer, Chemoresistance, Preoperative chemotherapy

## Abstract

**Background:**

Emerging evidence suggests molecular and phenotypic association between treatment resistance and epithelial–mesenchymal transition (EMT) in cancer. Compared with the well-defined molecular events of miR-200a in EMT, the role of miR-200a in therapy resistance remains to be elucidated.

**Methods:**

Breast cancer cells transfected with mimic or inhibitor for miR-200a was assayed for chemoresistance in vitro. miR-200a expression was assessed by quantitative real-time PCR (qRT-PCR) in breast cancer patients treated with preoperative chemotherapy. Luciferase assays, cell proliferation assay were performed to identify the targets of miR-200a and the mechanism by which it promotes treatment resistance. Survival analysis was used to evaluate the prognosis value of miR-200a.

**Results:**

In this study, our results showed ectopic expression of miR-200a promotes chemoresistance in breast cancer cell lines to several chemotherapeutic agents, whereas inhibition of miR-200a enhances gemcitabine chemosensitivity in resistance cancer cells. We found overexpression of miR-200a was closely associated with poor response to preoperative chemotherapy and poor prognosis in breast cancer patients. Furthermore, knockdown of YAP1 and TP53INP1 phenocopied the effects of miR-200a overexpression, and confirmed that TP53INP1 is a novel target of miR-200a. Remarkably, TP53INP1 expression is inversely correlated with miR-200a expression in Breast cancer cell lines. Taken together, these clinical and experimental results demonstrate that miR-200a is a determinant of chemoresistance of breast cancer.

**Conclusions:**

Upregulated miR-200a enhances treatment resistance via antagonizing TP53INP1 and YAP1 in breast cancer.

**Electronic supplementary material:**

The online version of this article (10.1186/s12885-017-3930-0) contains supplementary material, which is available to authorized users.

## Background

Breast cancer is one of the leading causes of cancer death among women. Although Exciting progress has been made with the chemotherapy of breast cancer, the development of chemoresistance remains a major obstacle to successful treatment of breast cancer [1]. A better understanding of the mechanism of chemoresistance in breast cancer is needed to offer more effective treatment and improve prognosis.

Accumulating evidence indicates that altered miRNA expression, such as miR-193a, miR-504 and miR-125b had been implicated in the response of tumor cells to chemotherapy and affected the sensitivity of cancer cells to treatment [2–4]. miR-200a is known to suppress Epithelial mesenchymal transition (EMT) [5–7]. Tumors undergoing EMT has been shown to resist conventional chemotherapy [8, 9]. The molecular events of miR-200a linking EMT are becoming well defined, while the role of miR-200a in therapy resistance remains unclear.

Our previous study has shown overexpression of miR-200a promotes anoikis resistance and metastasis in human breast cancer. In present study, we found TP53INP1 as a novel target of miR-200a. We show that TP53INP1 and YAP1 are direct targets of miR-200a that contribute to the ability of miR-200a to promote chemoresistance. In summary, we combined the results to establish a role for miR-200a in chemoresistance.

## Materials and methods

### Cell lines and breast tumor specimens

The cell lines MDA-MB-231 (HTB-26™), MDA-MB-436 (HTB-130™), MDA-MB-453 (HTB-131™), MDA-MB-468 (HTB-132™), BT-549 (HTB-122™), ZR-75-1 (CRL-1500™), ZR-75-30 (CRL-1504™), T47D (HTB-133 ™), MCF-7 (HTB-22™), SK-BR-3 (HTB-30™) and MCF-10A (CRL-10317™) were obtained from American Type Culture Collection (ATCC) and maintained in complete growth medium according to the ATCC-recommended culture conditions. HBL-100 was obtained from the Shanghai Cell Bank Type Culture Collection Committee (CBTCCC, Shanghai, China) and cultured in DMEM (Gibco, Gaithersburg, MD, USA) supplemented with 10% fetal bovine serum (FBS) (Gibco). Mycoplasma testing was routinely conducted by HD Biosciences. All cells were stored in liquid nitrogen and used for no more than 6 months after being thawed.

Patients with locally advanced breast cancer (>5 cm in diameter or with clinically palpable axillary adenopathy) received preoperative chemotherapy, weekly paclitaxel (80 mg/m2) and carboplatin (AUC = 2). All human breast cancer specimens were obtained from patients who were diagnosed with breast cancer and underwent surgery at the Shanghai Cancer Center during 2003–2009 (*n* = 110). All specimens contained over 90% tumor cells and were stored in liquid nitrogen until analysis. The study was approved by the Ethics Committee of the Cancer Hosipital, Fudan University and each patient gave written informed consent to participation in this research.

### siRNA, miRNA, plasmid construction, transfection, and luciferase assays

Specific siRNAs and scrambled siRNA were purchased from Genepharma, and miR-200a mimics, antagomiRNA, and negative controls were obtained from Ribobio. MicroRNA-200a represents miR-200a-5p in the current study. Transfection of siRNA were same as previously described [10]. The negative controls had no detectable effects in human cell lines or tissues (Additional file 1: Table S1).

The 3’-UTR of human TP53INP1 mRNA was cloned into the XhoI/NotI sites of the psiCHECK2 vector (Promega) using the In-Fusion® Advantage PCR Cloning Kit (Clontech). Luciferase assays were conducted as we previously reported [10]. The primers are listed in Additional file 1: Table S1.

### Immunoblotting

Immunoblotting was performed using a standard method [10]. Primary antibodies used were: anti-p73, anti-cleaved caspase-3, and anti-Bim (Epitomics); anti-Noxa (AbD Serotec); anti-YAP1, anti-Bax, and anti-GAPDH (Proteintech); and anti-TP53INP1 (Prosci). Secondary antibodies used were horseradish peroxidase-conjugated anti-rabbit and anti-mouse IgG (Proteintech). Quantity One software (BioRad) was used to quantify protein expression.

### RNA extraction and quantification of miRNAs

The tissues were homogenized with Polytron PT100. Total RNA was extracted using the Trizol reagent (Invitrogen), according to the manufacturer’s instructions. cDNA was synthesized from total RNA using a specific stem-loop RT primer (50 nM) with the ReverTra Ace®qPCR RT Kit (Toyobo). The primers for miR-200a and U6 detection assays were purchased from Ribobio. Real-time PCR was performed using the SYBR® Green Real-time PCR Master Mix (Toyobo) and conducted on the 7900HT Fast Real-Time PCR System (Applied Biosystems). The relative amount of miRNA or mRNA in each sample was calculated using the comparative CT method as described in our previous study [10].

### Establishment of gemcitabine resistant cell line

MDA-MB-231 cells were selected using 1 μM gemcitabine for 24 h, and then they were washed with PBS and recultured in drug-free medium. The treated cells were pulsed with 1 μM gemcitabine during periods of exponential growth. This cycle of treatment was repeated six times. The cell lines were cultured in 7 μM gemcitabine to generate gemcitabine-resistant sublines. Prior to the start of the experiments, the MDA-MB-231 GR cells were cultured in a gemcitabine-free medium.

### TUNEL assays

Synthetic miR-200a- and miR-control-transfected cells were plated onto poly-lysine-treated cover clips in six-well plates. Then, cells were treated with 5 μM cis-platin for 24 h. Apoptosis was detected using the in situ cell death detection kit with TMR red (Roche). Phalloidin-TRITC (10 μg/ml) (Sigma) was used to stain for actin, and DAPI was used to stain the nucleus. Confocal microscopy was then used to analyze the results.

### Proliferation assays

Cells were plated in 96-well plates. Following a 12-h culture, drugs were added. Each condition was repeated in triplicate, and untreated cells served as controls. After treatment for 72 h, 10 μl per well of WST-8 (Dojindo) was added to analyze cell viability. After culturing for 3 h, the absorbance at 450 nm was recorded using a 96-well plate reader. The growth inhibition ratio was calculated as follows: (%) = (OD. control well – OD. treated well) / OD. control well *100%.

### miRNA targets prediction

The following online software programs were used: TargetScan 5.2, and BioGRID 3.1 [11, 12]. The predicted targets of miR-200a, including the p53 family members as well as binding proteins that may regulate the p53 pathway were exported into Cytoscape and analyzed for evidence of intersection [13].

### Statistical analysis

The results of at least 3 experiments are expressed as the mean ± SD. An ANOVA and the Student’s t test were used to compare values between the test and control samples. Fisher’s exact χ2 test was used for categorical patient variables. Statistical significance was represented as *P* values <0.05. The optimal cut-off value for miR-200a expression was calculated by a X-tile program [14]. All statistical calculations were performed using STATA software.

## Results

### miR-200a plays a role in chemoresistance of breast cancer cells

**Fig. 1:**
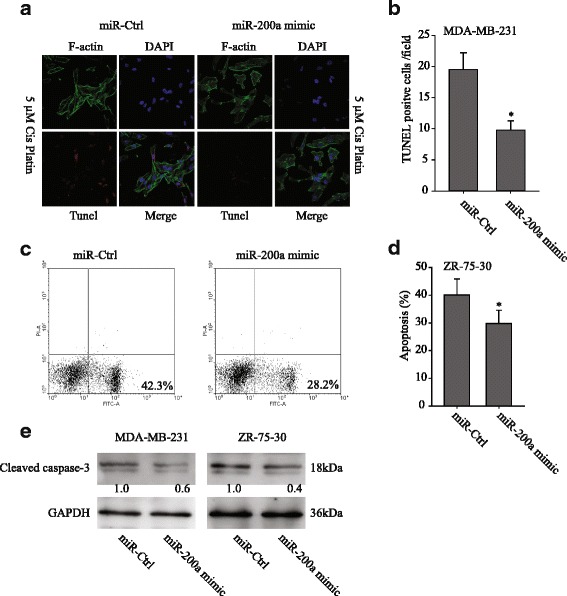
miR-200a promoted chemoresistance in breast cancer cell lines. **a** MDA-MB-231 cells that had been transfected with the miR-200a mimic or the miR-Ctrl were treated with 5 μM cis-platin and stained with TUNEL-TMR red, phalloidin-FITC for actin and DAPI for the cell nucleus. **b** The confocal TUNEL analysis showed that the miR-200a-transfected MDA-MB-231 cells had lower levels of apoptosis than the miR-Ctrl-transfected MDA-MB-231 cells. **c** Apoptosis was evaluated in ZR-75-30 cells after treating with cis-platin and staining with Annexin-V at 48 h. The flow cytometry profile depicts Annexin-V-FITC staining on the *x*-axis and PI staining on the *y*-axis. The number represents the percentage of early apoptotic cells in each condition, and (**d**) Mean ± SEM of apoptotic cells from three different experiments. **e** After cis-platin treatment, the transfected cells were lysed for western blotting. The protein levels of cleaved caspase-3 were normalized to GAPDH

In our previous studies, it has been shown that MDA-MB-231 and ZR-75-30 had low levels of miR-200a. To further examine the role of miR-200a in chemosensitivity, MDA-MB-231 and ZR-75-30 cells were transfected with miR-200a mimics, respectively. The transfected cells were treated with 5 μM cis-platin for apoptosis assays. First, the confocal TUNEL analysis showed that the miR-200a-transfected MDA-MB-231 cells had lower levels of apoptosis than the miR-Ctrl-transfected MDA-MB-231 cells (Fig. 1a, b, and *p* < 0.05). Second, Annexin V/PI staining showed that ZR-75-30-miR-200a mimic cells were resistant to cis-platin-induced apoptosis. In contrast, the ZR-75-30-miR-Ctrl cells were sensitive to cis-platin-induced apoptosis (Fig. 1c, d, and *p* = 0.009). Third, cleavage of Caspase-3 was suppressed in both miR-200a mimic transfected cells compared with the miR-Ctrl transfected cells (Fig. 1e). Together, these data suggest that miR-200a plays a role in chemoresistance.

### miR-200a expression promotes chemoresistance in breast cancer cell lines

**Fig. 2:**
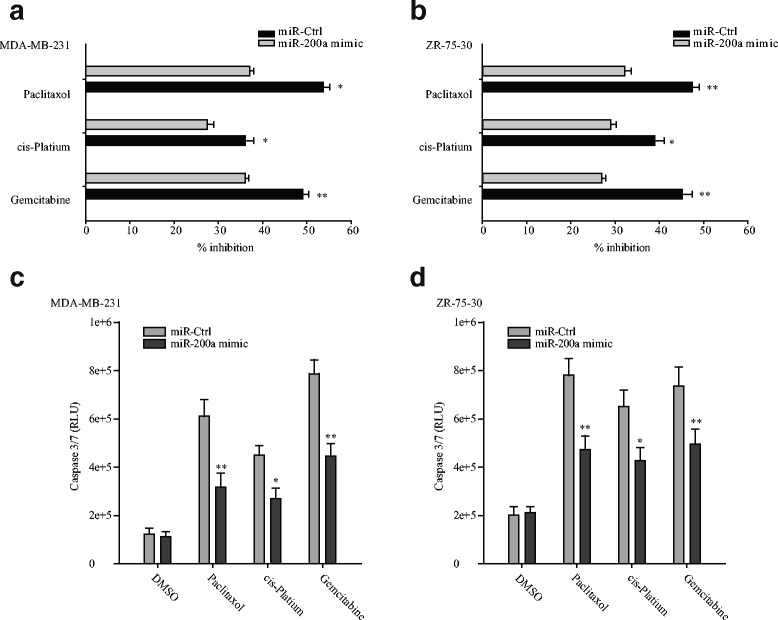
Ectopic expression of miR-200a promoted chemoresistance in breast cancer cell lines to multiple agents. Inhibition ratio of miR-200a and miR-Ctrl transfected MDA-MB-231 (**a**) and ZR-75-30 (**b**) after treating with chemo drugs 72 h. The drug concentrations were gemcitabine 5 μM, cis-platin 5 μM, and paclitaxel 60 nM, respectively. And the lysates were collected from MDA-MB-231(**c**) and ZR-75-30 (**d**) cells transfected with miR-200a and miR-Ctrl to analyze the activation of Caspases-3 and -7 using the Caspase GLO assay system. Data are mean ± SD of triplicate experiments. *p < 0.05; **p < 0.01

To examine the effects of miR-200a on chemosensitivity, we stably expressed miR-200a in the human breast cancer cell lines MDA-MB-231 and ZR-75-30. After treating with paclitaxel, cis-platin and gemcitabine, cells viability and Caspase 3/7 activity of stably miR-200a and scramble transfected cells were tested. In the cells viability assay, miR-200a overexpression significantly decreases the inhibitory effect of these chemotherapeutic agents. Furthermore, caspase3/7 activity assays showed that expression of miR-200a renders MDA-MB-231 and ZR-75-30 cells resistant to paclitaxel, cis-platin and gemcitabine induced apoptosis (Fig. 2a and b). On the other hand, scramble transfected cells were sensitive to apoptosis induced by these chemotherapy drugs (Fig. 2c and d). These results demonstrate a critical role for miR-200a in the chemoresistance of breast cancer cells.

### Overexpression of miR-200a induced chemoresistance was mediated through TP53INP1 and YAP1

**Fig. 3:**
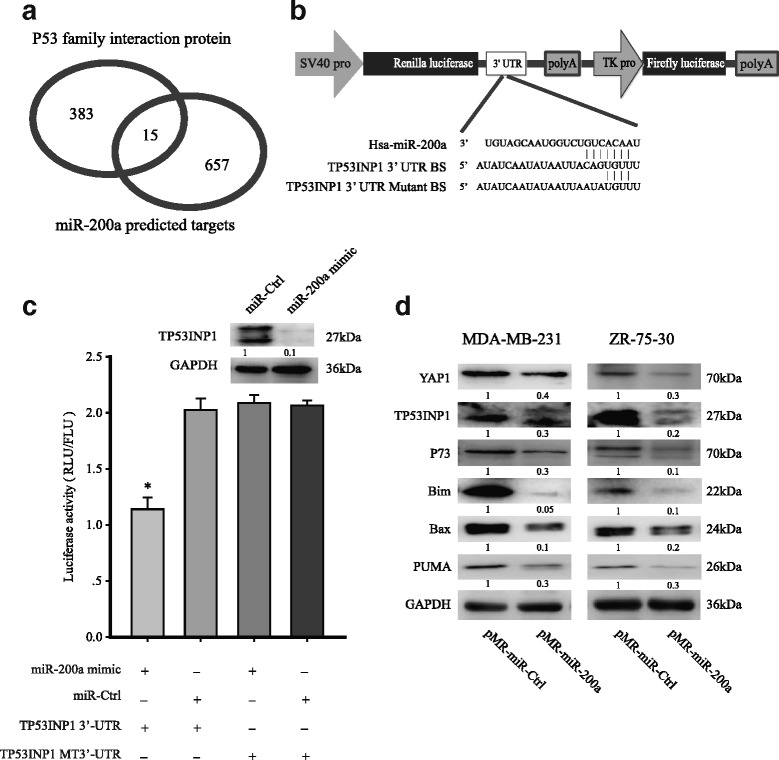
Overexpression of miR-200a induced chemoresistance was mediated through TP53INP1 and YAP1. **a** The Cytoscape map shows the intersection between the miR-200a predicted targets and the p53/p73 interacting proteins. **b** Sequence alignment of human miR-200a within the 3’-UTRs of TP53INP1. The seed sequence of miR-200a matches the 3’-UTRs of TP53INP1. Mutations within the 3’-UTRs of TP53INP1 in the mutant luciferase reporter constructs are as shown. The psiCHECK2-TP53INP1 vector was used for the luciferase assays. **c** 293 T cells were transfected with the indicated plasmids and oligonucleotide. Firefly luciferase activity was normalized with Renilla luciferase activity. Relative luciferase activities are presented. Data represent three independent experiments in triplicate. **d** The expression of TP53INP1, YAP1 and p73 were evaluated using western blotting on samples from pMR-miR-200a and pMR-miR-Ctrl transfected MDA-MB-231 cells. GAPDH was used as a control. Downstream effectors of p73, including BAX, PUMA, and Bim, were also detected by western blotting. The data were derived from three replicated experiments. *p < 0.05

To elucidate the mechanism behind the chemoresistance induced by miR-200a, we used the BioGRID and TargetScan databases (http://www.targetscan.org/) to search for potential targets of miR-200a [12]. Cytoscape was used to find intersection between predicted miR-200a targets and p53 family binding proteins responsible for the regulation of p53, p63 and p73 (Fig. 3a, Additional file 1: Table S2) [13]. We found that TP53INP1 and YAP1 are involved in p73-mediated apoptosis pathway and have a high degree of complementarity with the seed region of miR-200a (Fig. 3b). To confirm whether miR-200a also directly regulates the expression of TP53INP1, the 3’ UTR region of the TP53INP1 was cloned downstream of the luciferase open reading frame to construct the reporter plasmid psiCHECK2–3’UTR-TP53INP1-luc (Fig. 3b). Transient transfection of 293 T cells with the reporters and a miR-200a mimic led to significantly decreased reporter activity compared to the transfection of control oligonucleotides. Importantly, the miR-200 mimic did not decrease the luciferase activity of a mutant construct that contained substitutions at three nucleotides within the miR-200a binding site (Fig. 3c). These results indicate that miR-200a downregulates TP53INP1 expression by directly targeting its 3’ UTR.

TP53INP1 and YAP1 are involved in the DNA damage-induced p73-mediated apoptosis. We further detected the expression proapoptotic proteins puma, Bax, bim and noxa in miR-200a transfected cell lines. Unexpectedly, in miR-200a transfected MDA-MB-231, the p73 pathway expression levels of YAP1 and TP53INP1 were low, which resulted in the transcriptional repression of the pro-apoptosis target genes puma, Bax, bim and noxa (Fig. 3d). No significant difference was detected in expression of p73 in mRNA level (Additional file 2: Fig. S1). These data suggest that miR-200a confer insensitivity to drug-induced apoptosis by antagonizing YAP1 and TP53INP1 expression.

**Fig. 4:**
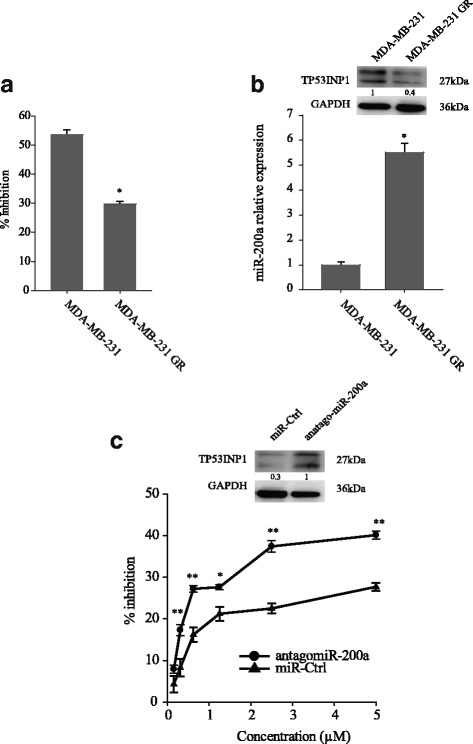
Inhibition of miR-200a restored the sensitivity to chemotherapy. **a** The gemcitabine-resistant MDA-MB-231 cells showed greater resistance to gemcitabine and a lower growth inhibition ratio. **b** The miR-200a level is increased in gemcitabine-resistant cells. **c, d** miR-200a inhibitor enhanced the sensitivity of gemcitabine-resistant cells to gemcitabine. Data are presented as mean ± SD from at least three separate experiments. *p < 0.05; **p < 0.01

In the next series of studies, we established the gemcitabine-resistant cell line (GR), MDA-MB-231 GR (Fig. 4a). qRT-PCR and western blot confirmed a significantly higher miR-200a expression in gemcitabine-resistant cell lines than in its parent cells (5.5-fold change of mRNA level in MDA-MB-231) (Fig. 4b). The treatment resistance of the MDA-MB-231 GR cell line was reversed by transfecting with the antagomiR-200a (Fig. 4c). Taken together, these data indicate that miR-200a is a determinant of chemosensitivity in breast cancer cells.

### Overexpression of miR-200a was associated with poor response to preoperative chemotherapy and poor prognosis in patients with breast cancer

To further define the clinical relevance of miR-200a and chemoresistance, the miR-200a expression patterns were also observed in human primary chemoresistance cancer tissues. The expression of miR-200a in preoperative chemotherapy treated breast cancer tissues were quantified by qRT-PCR. In Table 1, we compared the relationship between miR-200a expression and clinicopathologic characters of patients received preoperative chemotherapy followed by surgery. Chemotherapy response evaluation was following the RECIST guideline (version 1.1) [15].

**Table 1:** The relationship between miR-200a expression and clinicopathologic parameters in patients with breast cancer received preoperative chemotherapy

Clinicopathologic parameters	Number of cases	Median expression of miR-200a (normalized C_T_)^a^	*P*
Age,y			0.731
≤ 40	13	10.60 ± 2.870	
> 40	59	10.36 ± 2.939	
Histological type			0.892
DCIS	3	10.63 ± 1.572	
IDC	69	10.39 ± 2.959	
Menstrual status			0.272
Menstrual	38	10.05 ± 2.514	
Menopause	34	10.81 ± 3.285	
TNM stage			0.918
II	28	10.45 ± 3.601	
III	44	10.38 ± 2.412	
Tumor size			0.839
T1 + T2	32	10.48 ± 2.639	
T3 + T4	40	10.34 ± 3.138	
Chemotherapy response			0.0021
PD + SD	22	8.958 ± 2.586	
PR	50	11.04 ± 2.835	
ER			0.277
Negative	27	9.920 ± 2.648	
Positive	45	10.69 ± 3.045	
PR			0.501
Negative	23	10.06 ± 2.166	
Positive	49	10.56 ± 3.206	
Her2			0.515
Negative	52	10.54 ± 3.049	
Positive	20	10.04 ± 2.541	
All cases	72	10.40 ± 2.908	

^a^ normalized C_T_ = C_T_ miR-200a – C_T_ U6

**Fig. 5:**
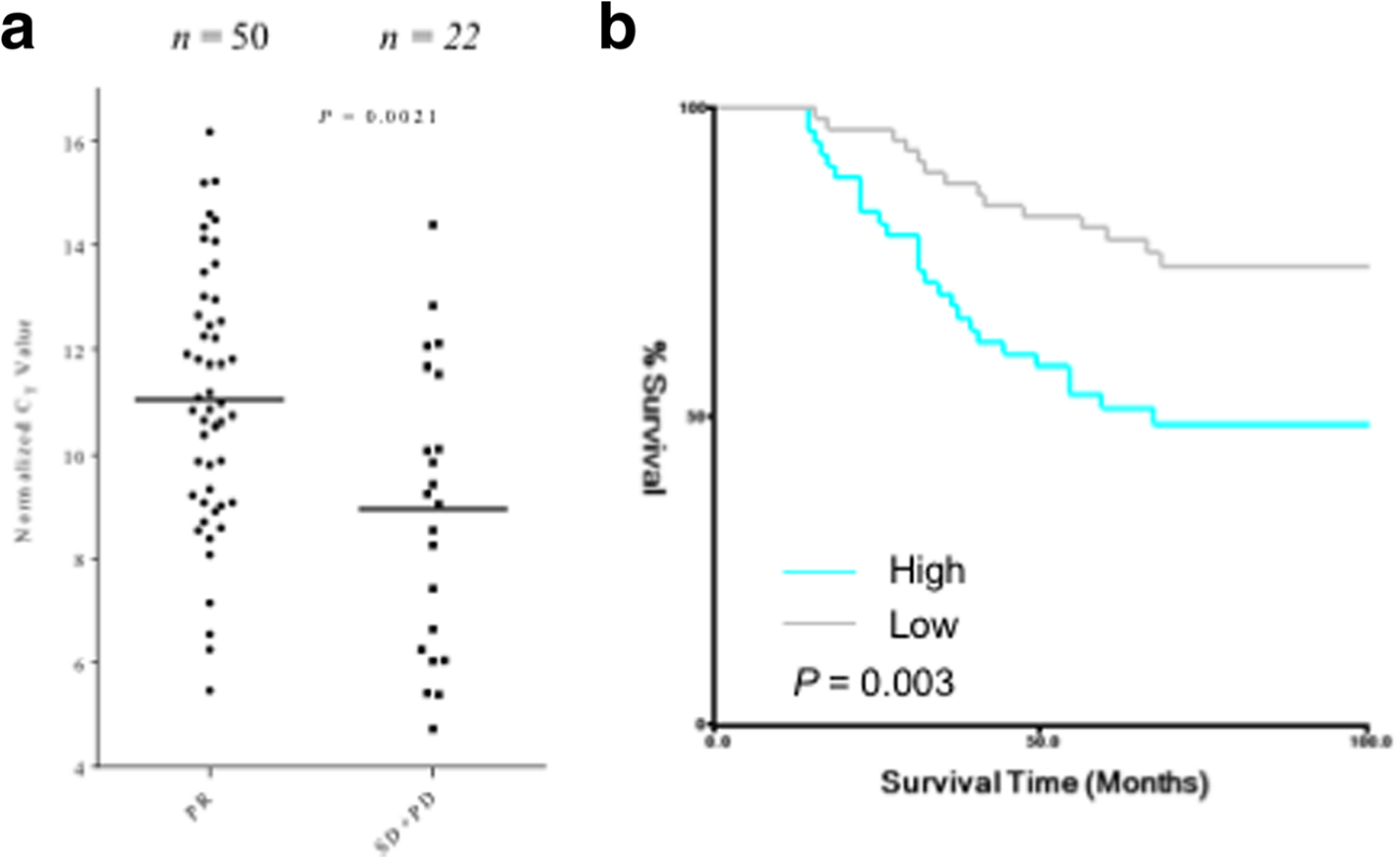
Overexpression of miR-200a was associated with poor response to preoperative chemotherapy and poor prognosis in patients with breast cancers. **a** Relationship between miR-200a expression and response to preoperative chemotherapy in patients with breast cancer. 72 patients with breast cancer who received preoperative chemotherapy followed by surgery were divided into two groups according to the chemotherapy response. The means of the two groups were significantly different from each other (*t*-test, *p* = 0.0021). **b** Kaplan-Meier curves of disease free survival rates of 110 patients with breast cancer who had definitive surgical treatment of their primary tumors according to miR-200a expression. The optimal cut-off point was determined by X-tile program. PR: partial response, SD: stable disease, PD: progressive disease

Significantly, the expression of miR-200a correlated inversely and significantly with the response to chemotherapy (Table 1; Fig. 5a; *p* = 0.0021). Furthermore, a survival analysis was performed to calculate the disease-free survival (DFS) for breast cancer patients (*n* = 110). The results showed that overexpression of miR-200a correlated significantly with shorter disease free survival duration of patients with breast cancer. (Fig. 5b). These results suggest the involvement of miR-200a overexpression in chemoresistance and poor prognosis.

### Knockdown of TP53INP1 and YAP1 phenocopied effects of overexpression of miR-200a

**Fig. 6:**
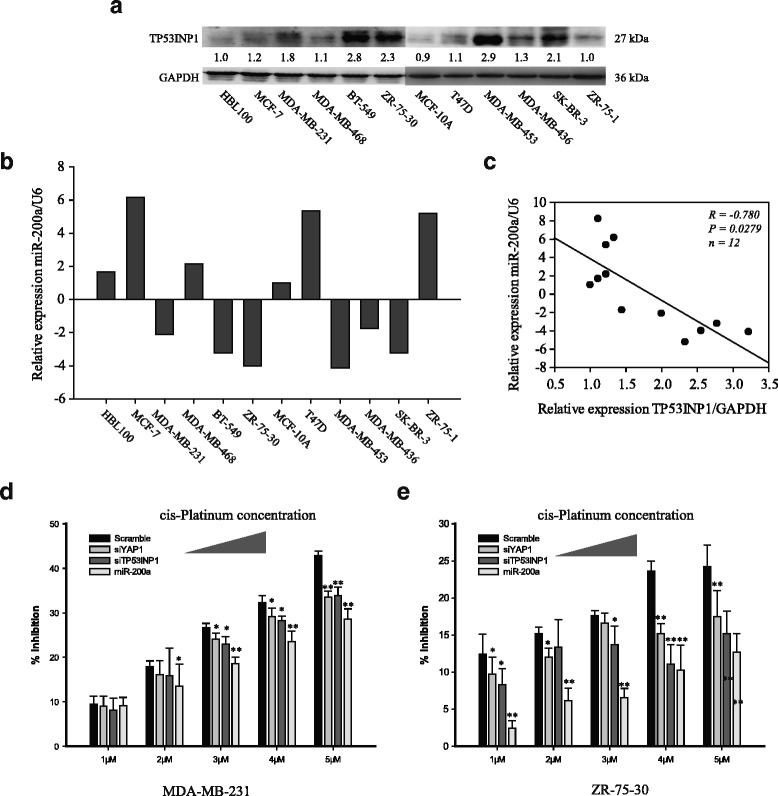
miR-200a expression is inversely correlated with TP53INP1 expression in breast cancer tissue. **a** Western blot analysis of TP53INP1 protein expression. **b** relative expression levels of miR-200a in human breast cancer cell lines. **c** Pearson correlation of the TP53INP1 and miR-200a expression levels in breast cancer cell lines (*R* = −0.780, *P* = 0.0279, *n* = 12). Inhibition ratio of scramble RNA, siYAP1, siTP53INP1 and miR-200a mimic transfected MDA-MB-231 (**d**) and ZR-75-30 (**e**) after treating with cis-platin 72 h. The cis-platin concentrations were 1 μM, 2 μM, 3 μM, 4 μM, and 5 μM, respectively. *p < 0.05; **p < 0.01

To further identify the association between TP53INP1 and miR-200a in breast cancer, the expression of miR-200a and TP53INP1 were examined in various breast cancer cell lines (Fig. 6a and b). A reverse correlation was observed between miR-200a expression and TP53INP1 expression. The cells overexpressing miR-200a had a lower level of TP53INP1 expression (*R* = −0.780, *p* = 0.0279, *n* = 12) (Fig. 6c). This data further indicated the functional link between miR-200a and TP53INP1 in breast cancer. Moreover, silencing TP53INP1 and YAP1 can partly reproduce treatment resistance of miR-200a overexpression (Fig. 6d and e). These results implied that, enforced expression of miR-200a in breast cancer cell lines conferred chemoresistance via targeting YAP1 and TP53INP1.

## Discussion

Despite recent advances in chemotherapy, the development of resistance to chemotherapy remains a major clinical issue. In this study, we demonstrate that miR-200a plays an important role in chemoresistance. The results indicated that high expression of miR-200a was closely associated with poor response to preoperative chemotherapy. In addition, the effects of miR-200a on chemoresistance were mediated through antagonizing TP53INP1 and YAP1. These findings demonstrate that overexpression of miR-200a in breast cancer is associated with chemoresistance.

Several miRNAs were known to be associated with chemotherapeutic efficacy. miR-504 and miR-125b can negatively regulate p53 protein level by directly binding to specific sites within the 3’ UTR of p53 mRNA [3, 4]. More recently, miR-193a, which is regulated by p63 and targets p73, was found to be a key regulator of p63/p73-dependent chemoresistance in squamous cell carcinoma [2]. In this study we showed that miR-200a expression is significantly associated with the response to chemotherapy in patients with breast cancer. Furthermore, we found miR-200a was overexpressed in gemcitabine resistant breast cancer cells, and inhibition of miR-200a can restore sensitivity to these cells. Unfortunately, we cannot determine the dynamic expression of miR-200a after treating with chemotherapy due to small chemotherapy samples and a relatively limited number of patients. Despite its preliminary character, this study clearly indicates that miR-200a acts as a negative regulator of chemotherapy induced apoptosis therefore confer chemoresistance.

**Fig. 7:**
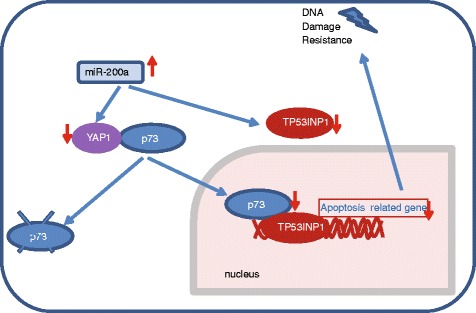
Schematic representation of the effects of miR-200a on chemoresistacne

The susceptibility of tumor cells to chemotherapy induced death is a major determinant in the outcome of therapy. p73 plays a central role in chemotherapy resistance; the regulatory mechanisms that control the p73 chemotherapy response are closely related to posttranslational modifications and protein-protein interactions [16]. In the current study, mRNA expression of p73 is not significantly changed with variation of miR-200a expression, indicating that regulation of p73 is not at RNA level but posttranslational level. In present study, the results showed that miR-200a can negatively regulate TP53INP1 expression through binding to its 3’ UTR of mRNA. TP53INP1, which is referred to as a stress-induced protein, can be transcriptionally activated by p53 or p73 and promote p53 phosphorylation at Ser-46 as well as apoptosis in response to DNA damage [17, 18]. We used MDA-MB-231 (TP53 mutant) and ZR-75-30 (TP53 wild-type) in the current study and miR-200a plays a role in mediating chemoresistance in both cell lines. We believe p53 does not matter in miR-200a mediated chemoresistance. TP53INP1 is a downstream effector of p73 in p53-mutant cell lines [19]. Our previous study has also shown that miR-200a functions as a repressor of YAP1, which stabilized p73 under conditions like DNA damage and stress [20–24]. Therefore, in response to chemotherapy, miR-200a may lead to p73 degradation, causing attenuated transcription of downstream proapoptotic genes, such as Bax, Bim and Puma. miR-200a may also prevent apoptosis by targeting TP53INP1 [25]. Thus, by targeting two nodes of the chemo-induced apoptosis pathway, miR-200a confers resistance to chemotherapy (Fig. 7).

## Conclusions

In summary, we established a role of miR-200a in chemotherapy resistance. We showed that miR-200a promotes DNA damage resistance by inhibiting DNA damage-induced apoptosis via YAP1 and TP53INP1 in breast cancer. Deciphering the association of miR-200a and chemoresistance might provide important insights into tumor progression and treatment.

## Additional files

Additional file 1: Table S1. List of primer and siRNA sequences. **Table S2**: Intersection between predict target of miR-200a and p53 family binding partner. (DOCX 17 kb)

Additional file 2: Fig. S1. Expression of p73 in MDA-MB-231 and MDA-MB-231 GR cells. (EPS 588 kb)

## Abbreviations

DFS: Disease-free survival
EMT: Epithelial-mesenchymal transition
GR: Gemcitabine-resistant
qRT-PCR: quantitative real-time PCR
