# Supplementary material for: MicroRNA-200a confers chemoresistance by antagonizing TP53INP1 and YAP1 in human breast cancer
Source: BMC Cancer. 2018 Jan 12;18:74. doi: 10.1186/s12885-017-3930-0 (PMC5766993; doi:10.1186/s12885-017-3930-0)
Supplement: Supplementary file 1 — List of primer and siRNA sequences. Table S2: Intersection between predict target of miR-200a and p53 family binding partner. (DOCX 17 kb) [file 12885_2017_3930_MOESM1_ESM.docx]

**Additional files**

Table S1. List of primer and siRNA sequences

| **Purposes** | **sequence type** | **Sequence** |
| --- | --- | --- |
| TP53INP1 3’ UTR | Sense | TTGCTAAGTAACTTTTCCAGTTTTG |
|  | Antisense | CCTACGTGTGAATCGAACCC |
| TP53INP1 3’ UTR mutant* | Sense | TCAGATTATTTCTGGAAACATATCAATATAATTAATATGTTTGGGGGTGTCTTTAAAT |
|  | Antisense | ATTTAAAGACACCCCCAAACATATTAATTATATTGATATGTTTCCAGAAATAATCTGA |
| psiCHECK2-TP53INP1 UTR** | Sense | TAGGCGATCGCTCGAGTAGTTTCAAGTTTTGTTGGTTG |
|  | Antisense | TTGCGGCCAGCGGCCGCCCTACGTGTGAATCGA |
| siRNA TP53INP1 464 | Target | CATAGATACTTGCACTGGTTT |
| siRNA TP53INP1 580 | Target | GCTTGGCTGATACAAGTGATT |
| siRNA negative control |  | GUGGAUAUUGUUGCCAUCA |

* Site mutation PCR primer ** Infusion PCR primer

Table S2. Intersection between Predict target of miR-200a and p53 family binding partner

|  | | | |
| --- | --- | --- | --- |
| **Number** | **Gene Name** | **Biological process*** | **Score**** |
|  | | | |
| **1** | CDC14A | Cell cycle, cell division | 99 |
| **2** | THRB | regulation of transcription from RNA polymerase II promoter | 97 |
| **3** | TFAP2C | Cell-cell signaling | 97 |
| **4** | SIRT1 | positive regulation of anti-apoptosis | 89 |
| **5** | YAP1 | cellular response to DNA damage stimulus | 88 |
| **6** | TP53INP1 | apoptosis | 81 |
| **7** | RYBP | histone H2A monoubiquitination | 79 |
| **8** | NFYB | Transcription regulation | 66 |
| **9** | PTEN | negative regulation of G1/S transition of mitotic cell cycle | 66 |
| **10** | TFAP2A | negative regulation of reactive oxygen species metabolic process | 58 |
| **11** | CDC42 | positive regulation of cell cycle cytokinesis | 48 |
| **12** | HIPK2 | DNA damage response | 45 |
| **13** | HNRNPAB | epithelial to mesenchymal transition | 40 |
| **14** | KPNA4 | NLS-bearing substrate import into nucleus | 31 |
| **15** | ZNF148 | cellular defense response | 17 |
|  | | | |

* Data from Protein knowledge database

** The potential targets were sequenced by Scores. The Scores were from TargetScan Human 5.2 conserved miRNA family context score.
